# Molecular characterization of measles viruses in China: Circulation dynamics of the endemic H1 genotype from 2011 to 2017

**DOI:** 10.1371/journal.pone.0218782

**Published:** 2019-06-20

**Authors:** Huiling Wang, Yan Zhang, Naiying Mao, Zhen Zhu, Aili Cui, Songtao Xu, Jinhua Song, Meng Chen, Xueqiang Fang, Chongshan Li, Daxing Feng, Shujie Zhou, Shulei Wang, Jing Shi, Yixin Ji, Lei Cao, Li Ren, Lingyu Gao, Wenbo Xu

**Affiliations:** 1 NHC Key Laboratory of Medical Virology and Viral Diseases (National Institute for Viral Disease Control and Prevention, Chinese Center for Disease Control and Prevention), WHO WPRO Regional Reference Laboratory of Measles/Rubella, Beijing, People’s Republic of China; 2 Beijing Center for Disease Control and Prevention, Beijing, People’s Republic of China; 3 Shandong Center for Disease Control and Prevention, Jinan, Shandong, People’s Republic of China; 4 Shanghai Center for Disease Control and Prevention, Shanghai, People’s Republic of China; 5 Henan Center for Disease Control and Prevention, Zhengzhou, Henan, People’s Republic of China; 6 Anhui Center for Disease Control and Prevention, Hefei, Anhui, People’s Republic of China; 7 Yantai Center for Disease Control and Prevention, Yantai, Shandong, People’s Republic of China; 8 Yizhuang Hosptial, Beijing, People’s Republic of China; Keele University Faculty of Natural Sciences, UNITED KINGDOM

## Abstract

Due to the Expanded Program on Immunization (EPI) and supplementary immunization activities (SIAs) in China, the incidence of measles in China has decreased extensively. The incidence reached its lowest levels in contemporary history in 2012 and 2017, with incidence rates of 4.6 and 4.3 per million population, respectively. However, more than 147,000 measles cases were reported from 2013 to 2016. Furthermore, the proportions of cases in infants < 8 months and adults have been increasing since 2013, representing a considerable challenge for measles elimination in China. A total of 14,868 measles viruses were isolated from confirmed measles cases from 2011 to 2017, of which 14,631 were identified as the predominant endemic genotype, H1; 87 were identified as genotype A viruses that were vaccine associated strains; and 150 were identified as non-H1 genotype viruses. The non-H1 genotype viruses included 62 D8 viruses, 70 D9 viruses, 3 D11 viruses, 14 B3 viruses, and 1 G3 virus, which were identified as imported or import-related viruses that caused sporadic cases or small outbreaks. Most of the transmission chains detected during the period 2011–2012 were interrupted and were followed by many new transmission chains of unknown origin that spread, causing a large measles resurgence in China during 2013–2016. After 4 years of measles resurgence and continuous implementation of the routine immunization program and SIAs, the population immunity reached a sufficiently high level to interrupt most of the transmission chains; only a few strains survived, which continued to be sporadically detected in China in 2017. In the present study, the results from the combined epidemiological and molecular virological data demonstrated the great progress towards measles elimination in China by the further analysis of circulation dynamics for the endemic H1 genotype measles virus from 2011 to 2017. And this study accumulated critical baseline data on circulating wild-type measles viruses in China and provides comprehensive information to the world. These comprehensive baseline data provide evidence to support measles elimination in the future, not only in China but also in other countries worldwide. In addition, the information will be very useful to other countries for tracing their sources of measles cases and for identifying transmission links, which can help prevent potential measles outbreaks.

## Introduction

Measles is a highly contagious disease characterized by generalized maculopapular rash, high fever, cough, coryza and conjunctivitis, and it is caused by the measles virus (MeV) [1]. The measles virus is a negative-sense, single-stranded RNA virus in the genus *Morbillivirus* within the family *Paramyxoviridae*. The measles virus is monotypic, but genetic variability exists among wild-type strains [2]. The World Health Organization (WHO) currently recognizes 8 clades (A-H) and 24 genotypes of measles virus based on sequence analyses of the carboxyl-terminal (C-terminal) region of the nucleoprotein (N) gene and the entire hemagglutinin (H) gene [3–6].

Before the introduction and widespread use of the measles vaccine, measles infection occurred across the globe. In 2017, despite the availability of a safe and effective vaccine, approximately 110,000 people died from measles globally, most of whom were children under 5 years of age [7]. The measles vaccine was introduced in China in 1965, and in 1978, the national Expanded Program on Immunization (EPI) was established. Under the EPI, a standard schedule for routine immunization was implemented, which included one dose of measles vaccine administered at 8 months of age [8]. A second routine dose of measles vaccine to all children at 7 years of age was introduced in 1986, and the schedule for the second dose was changed to those aged between 18 and 24 months in 2005 [9]. China has made considerable progress towards the goal of measles elimination in recent years [8, 9]. To reach this goal, in addition to routine measles vaccination, supplementary immunization activities (SIAs) are conducted in China. Between 2004 and 2009, 27 of the 31 mainland provinces conducted unsynchronized province-specific measles SIAs targeting children aged 8 months through 14 years. In September 2010, China conducted nationwide synchronized SIAs targeting different age groups spanning 8 months through 14 years in different provinces [10, 11]. With the wide use of measles-containing vaccines, the incidence of measles in China has decreased extensively, reaching its lowest levels in history in 2012 and 2017. However, re-emergence of measles occurred in 2013 and continued from 2014 to 2016.

Genetic analysis of circulating wild-type viruses together with epidemiological information is a critical component of measles surveillance because it can help confirm the source of a virus or suggest a source for unknown-origin cases as well as establish links, trace the transmission pathway, and classify suspected cases as vaccine reactions [5]. Acquiring genotype information of circulating measles viruses is one of three essential criteria for verifying the progress, achievement and maintenance of measles elimination [5, 6]. In addition, imported measles virus strains can be identified in a timely manner through molecular characterization, thereby providing data to support the development of strategies to interrupt the spread of imported measles virus [4–6].

This study was conducted to describe the progress towards measles elimination in China based on an analysis of the epidemiology of measles and the molecular virological data of measles virus strains throughout China from 2011 to 2017. The results demonstrated the great progress towards measles elimination in China by further analysis of circulation dynamics for the endemic H1 genotype measles virus circulating in China.

## Materials and methods

### Source of measles epidemiological data

The number of measles cases, the annual incidence rates and the age breakdown of measles cases were taken directly from reports of the National Notifiable Disease Reporting System (NNDRS) [9], Chinese Center for Disease Control and Prevention (China CDC).

### Specimen collection and viral isolation

The China measles laboratory network was established in 2001 and comprises tiered structured laboratories. Currently, it includes one national measles laboratory, 32 provincial measles laboratories (including one laboratory of Xinjiang Construction Corps) and 339 prefecture laboratories [12]. Different tiers of laboratories have their own responsibilities: briefly, the national laboratory serves as the national reference standard to provide technical advice and support to provincial laboratories, whereas provincial laboratories have a specific virus isolation capacity, and prefecture laboratories have the responsibility of preliminarily diagnosing suspected cases. Throat swab and urine samples were collected from individuals suspected of having measles from 30 provinces in China through the national measles surveillance program. All clinical samples were collected within five days of rash onset and transported in accordance with standard protocols [13]. Isolation of measles virus was performed using the Vero/hSLAM cell line in provincial CDC, and infected cells were harvested when the cytopathic effect (CPE) was visible over at least 75% of the cell layer. The isolates were shipped to the China CDC for further analyses.

### Ethics statement

In this study, 14,868 sequences from more than 160,000 measles cases were analyzed, and only age information of the corresponding measles subjects was used in this study. Other subject information, such as name, gender and home address, were not included in our study. Therefore, the requirement for informed consent was waived by the ethics committee of National Institute for Viral Disease Control and Prevention of the China CDC. In this study, the only human materials used were throat swabs and urine specimens collected from clinically suspected measles patients for the purpose of public health and disease control. This study was approved by the second session of the Ethics Review Committee of the National Institute for Viral Disease Control and Prevention of the China CDC, and the methods were performed in accordance with the approved guidelines.

### RNA extraction and RT-PCR

According to the national measles surveillance guidelines of China, the provincial measles laboratories performed the virus isolation and shipped the isolates to the China CDC for further genotyping identification. If isolates were not obtained, then N450 sequences were obtained directly from clinical specimens for further genotyping. RNA was extracted from clinical specimens or viral isolates using the QIAamp Viral RNA Mini Kit (QIAGEN, Beijing, China) according to the manufacturer’s instructions. RT-PCR amplification was performed using previously described primers to amplify a 600-bp fragment in the N gene, which included the 450-bp fragment recommended for genotyping [11]. The PCR products were purified using the QIAquick Gel Extraction Kit (QIAGEN).

### Sequence analysis

Sequences of the PCR products were obtained using BigDye terminator chemistry version 3.0 according to the manufacturer’s protocol for both sense and antisense strands on an automated ABI PRISM 3100 DNA Sequencer (PerkinElmer, Beijing, China). Sequences were analyzed using Sequencer (Gene Codes Corporation, Ann Arbor, MI, USA) and version 7.0 of BioEdit (www.mbio.ncsu.edu/BioEdit/BioEdit.html). Phylogenetic analyses were performed and trees were generated using the Molecular Evolutionary Genetics Analyses (MEGA) software version 6. Phylogenetic trees were constructed by comparison with the reference strains defined by the WHO using the neighbor-joining method. The reliability of the groupings was estimated using bootstrap resampling of 500 replicates. Nucleotide sequence data from 152 representative strains were deposited in GenBank under the following accession numbers: MH979841-MH979992 (S1 Table).

## Results

### Epidemiological characterization of measles cases

**Fig 1:**
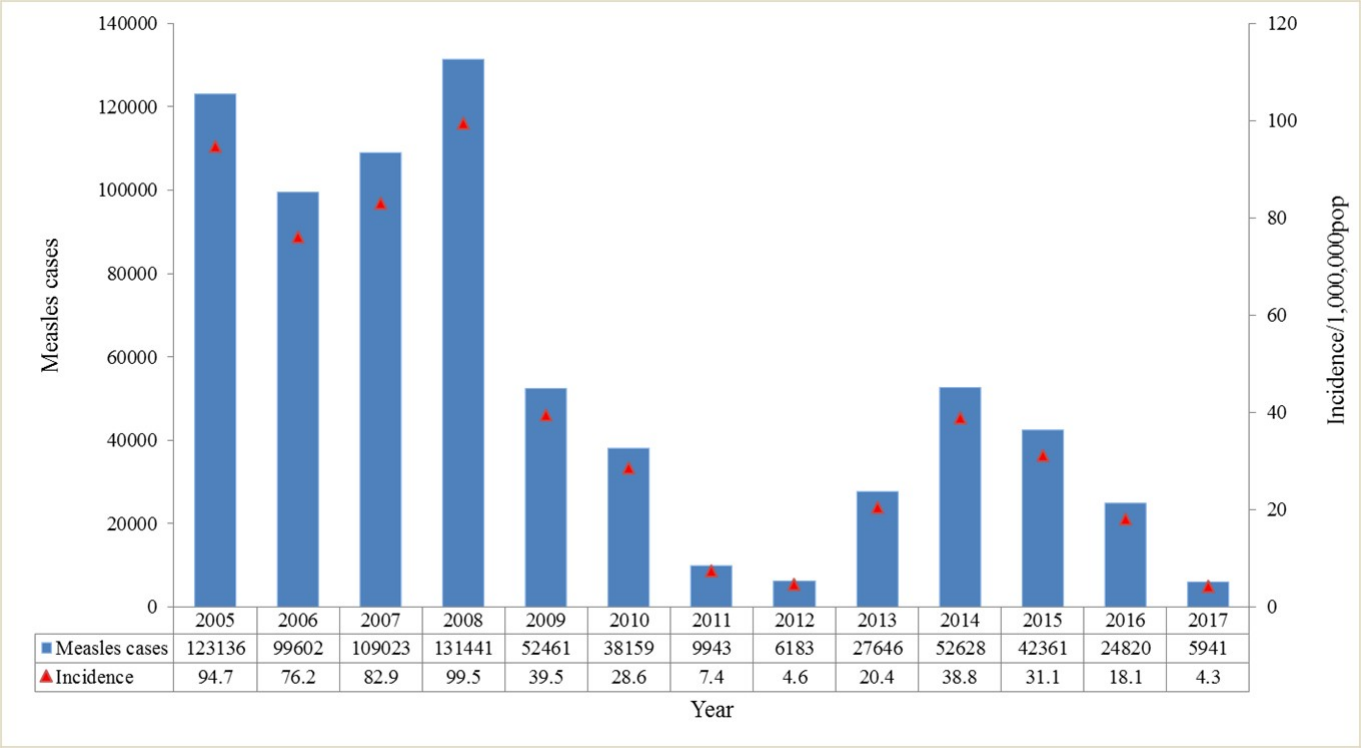
Number of measles cases and incidence from 2005 to 2017. Source: National Notifiable Disease Reporting System (NNDRS).

The annual reported measles cases decreased from 131,441 in 2008 to 6,183 in 2012, with a decrease in incidence from 99.5 to 4.6 per million population. However, an increase was observed from 2013 to 2016, with the number of reported cases ranging from 24,820 to 27,646 (and incidence ranging from 18.1 to 20.4 per million population), whereas the incidence reached the lowest level in history in 2017, with 5941 cases (4.3 per million population) (Fig 1).

**Fig 2:**
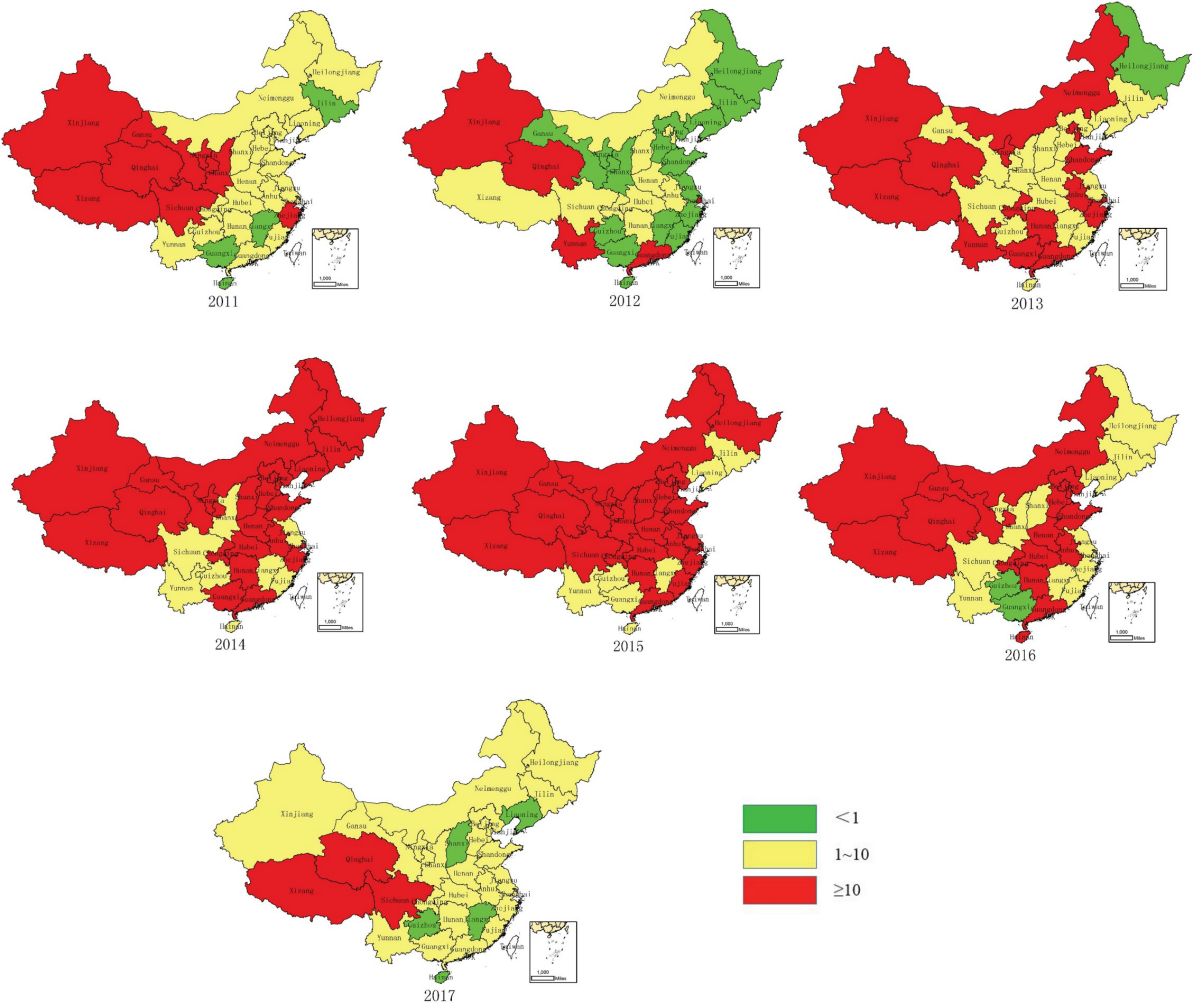
Measles incidence in China 2011–2017 by province. Source: NNDRS.

In 2012, the measles incidence was lower than one per million population in 15 provinces. However, measles resurgence occurred in many provinces during 2013–2016. The incidence was higher than 10 per million population during 2013–2016 in 14–24 provinces, with peaks occurring in 2015 (24 provinces). In 2017, four provinces achieved incidence rates of less than one per million population, 24 provinces achieved incidence rates between 1–10 per million, and three provinces continued to have high incidence rates of higher than 10 per million (Fig 2).

**Fig 3:**
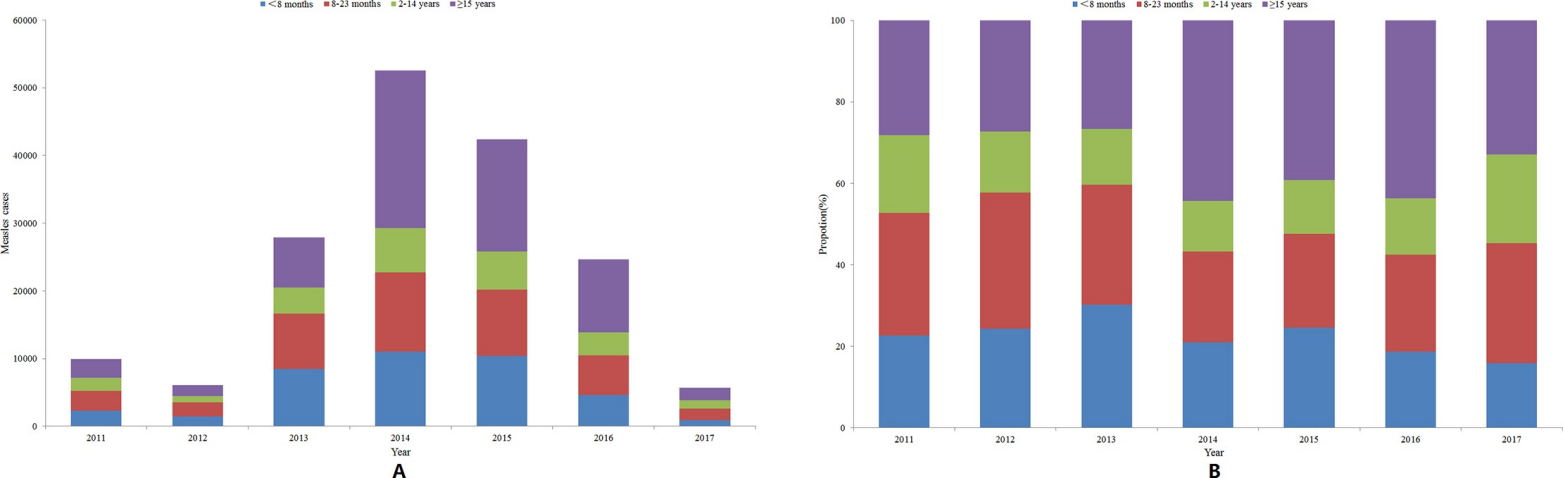
Measles cases in different age groups in China from 2011 to 2017. A: The number of measles cases in different age groups. B: The proportion of different age groups of measles cases. Source: NNDRS.

The age breakdown of measles cases from 2011 to 2017 is shown in Fig 3. The proportion of measles cases among those aged 8 months to 14 years decreased slightly over time from 50% in 2011 to 35% in 2016. The percentage of cases in children under 8 months old accounted for more than 20% of the cases in 2011, 2013, and 2015. Since 2014, the number of adult cases has increased: cases in patients greater than 15 years of age accounted for 44% of all cases in 2014 and 2016, 39% in 2015, and 33% in 2017 but only approximately 30% in each year before 2013.

### Measles virus genotypes

**Fig 4:**
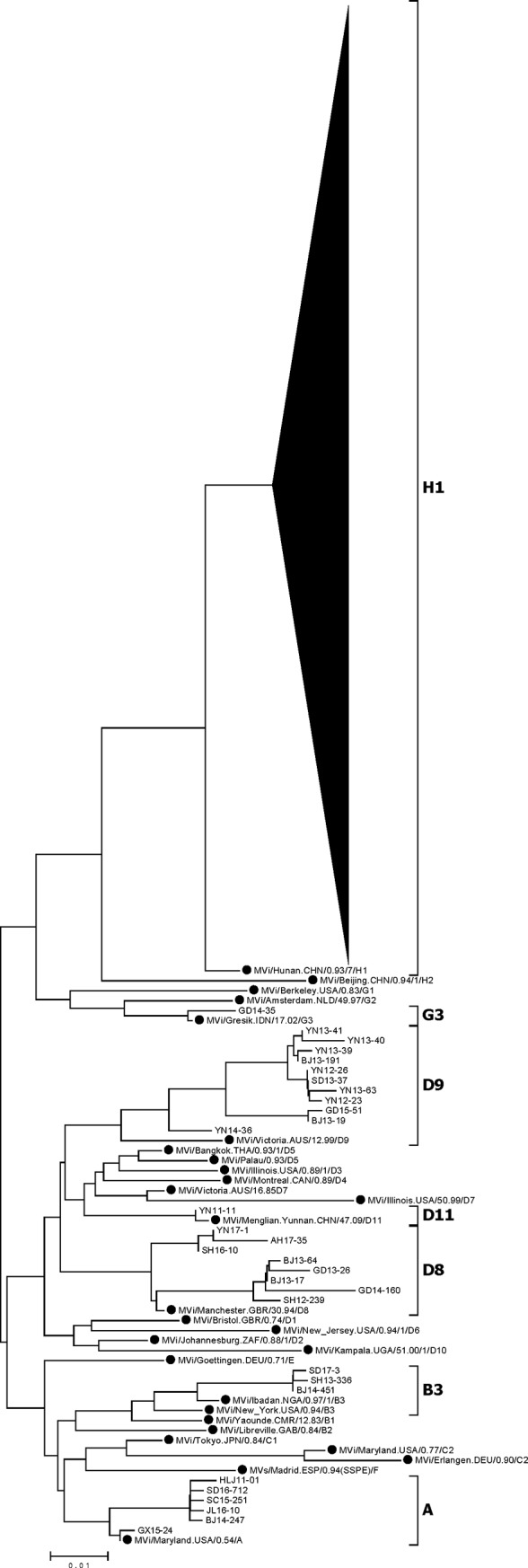
Phylogenetic tree of representative measles virus strains isolated in China from 2011 to 2017 and WHO reference measles virus strains constructed based on the 450-nucleotide sequence of the N gene C-terminal region. Significant bootstrap values (>90) are indicated. WHO reference strains are indicated by black dots.

In this study, 14,868 sequences of N450 from 5176 clinical specimens and 9692 viral isolates were obtained from 30 out of 31 provincial measles laboratories (i.e., all except that in Tibet) from 2011 to 2017. The phylogenetic analysis indicated that 14,631 (98.4%) sequences belonged to endemic genotype H1 (Fig 4), 87 to genotype A, 62 to D8, 70 to D9, 14 to B3, 1 to G3, and 3 to D11 (Table 1). All 87 A genotype viruses were isolated from vaccinated individuals and were associated with vaccine-related cases. A total of 294 and 474 strains were obtained in 2011 and 2012, respectively, whereas the annual number of obtained strains ranged from 2327 to 4915 from 2013 to 2016, with a peak in 2014. In 2017, following a large drop in measles cases, far fewer strains (421) were obtained. To illustrate the genotype identification clearly, representative strains were selected to construct a phylogenetic tree, as too many sequences were obtained in this study to be viewed in one figure. Identical sequences and sequences that had one nucleotide difference were excluded from the alignment, and the remaining 523 representative strains were selected from the 14,868 MeV strains and used together with the 24 WHO reference strains to construct a phylogenetic tree, which is shown in Fig 4.

**Table 1:** Numbers of measles viruses isolated from 2011 to 2017 by genotype.

Year	Genotype						
	H1	G3	D11	D9	D8	B3	A (VAC)
2011	288		3				3
2012	454			13	1		6
2013	2217			47	45	3	15
2014	4868	1		9	3	10	24
2015	3952			1			16
2016	2455				3		10
2017	397				10	1	13
**Total**	**14631**	**1**	**3**	**70**	**62**	**14**	**87**

### Phylogenetic analysis of measles viruses of the predominant endemic genotype H1

**Fig 5:**
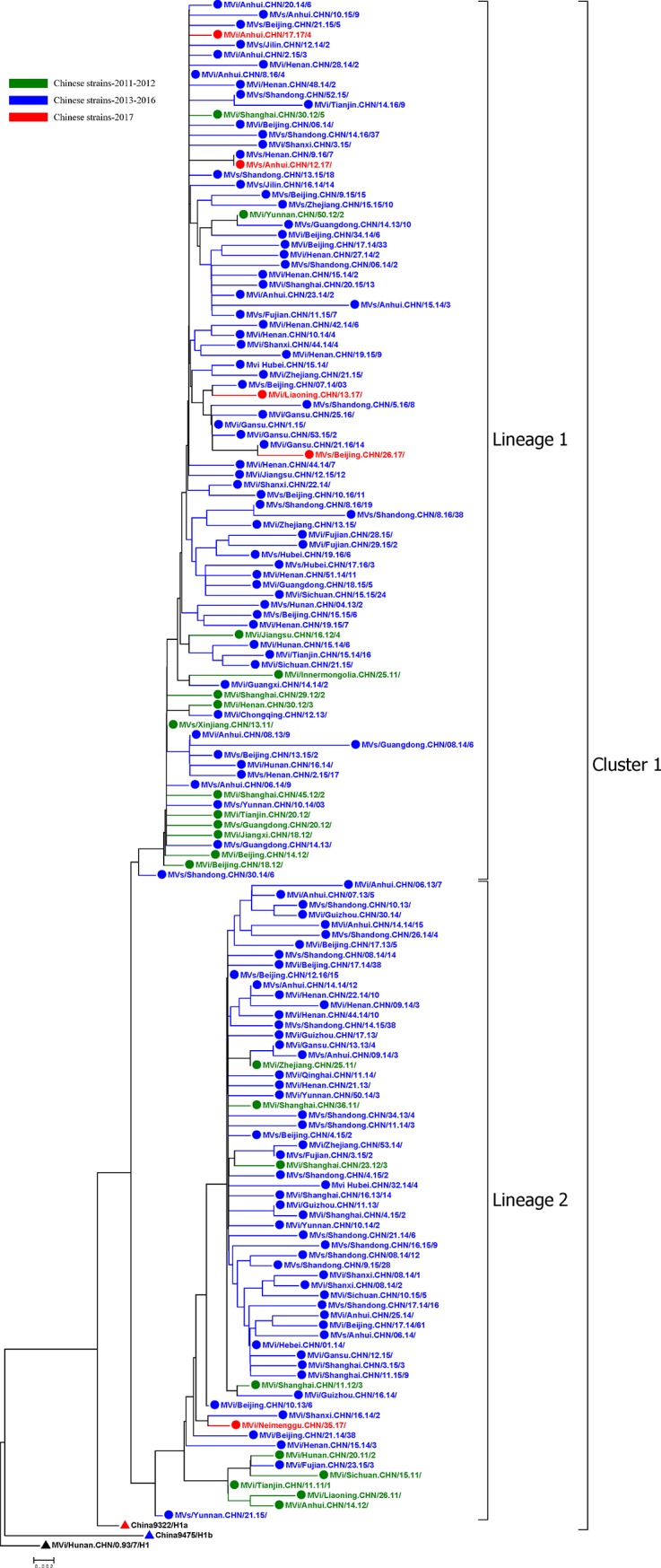
**Phylogenetic tree of representative wild-type measles virus strains in China during 2011–2017, a WHO reference strain of genotype H1 (black triangle), a reference strain of H1 genotype cluster 1 (red triangle), and a reference strain of H1 genotype cluster 2 (blue triangle) based on the 450-nucleotide sequence of the N gene C-terminal region.** Significant bootstrap values (>90) are indicated.

As with the preceding phylogenetic analysis, identical H1 sequences and H1 sequences from the same year that differed by only one or two nucleotides were excluded from the alignment, and the remaining 152 H1 representative strains were construct a phylogenetic tree (Fig 5). All of the 14,631 H1 sequences belonged to cluster 1 of genotype H1, and two main lineages within this cluster were observed. Multiple transmission chains existed within each lineage, without any apparent temporal or geographic pattern.

Viruses in the same transmission chain have identical or nearly identical N450 sequences [14]. In this study, a transmission chain was defined as viruses with identical or nearly identical (within two nucleotide differences) N450 sequences, as epidemiologic information was limited. Most of the transmission chains in 2011–2012 (shown in green in Fig 5) were not detected in 2013–2016. Therefore, we considered the transmission chains in 2011–2012 to have been interrupted. Many new transmission chains of unknown origin occurred in 2013–2016 (shown in blue in Fig 5) and were identified as new transmission chains associated with measles resurgence. After 4 years of measles resurgence, only a few strains persisted, being sporadically detected in China in 2017. The ranges of nucleotide sequence and amino acid homologies among the 14,631 H1 isolates were 95.7–100% and 92.6–100%, respectively. Compared with the WHO genotype H1 reference strain, the homology of the nucleotide sequences ranged from 96.2–98.2%, and that of the amino acid sequences ranged from 93.2–97.9%. When the sequences were BLASTed against those in the GenBank and WHO MeaNS (http://www.who-measles.org/) databases, we found that H1 viruses had been detected in other countries between 2011 and 2017, including, the USA, Australia, Japan, Mongolia, the United Kingdom, Thailand, and et al.

## Discussion

As a result of the EPI and SIAs in China, the incidence of measles in China has decreased extensively and reached a very low level in 2012. However, measles resurgence occurred during 2013–2016. With the continuous, routine immunization programs and SIAs, the population immunity reached a level sufficient to interrupt most of the transmission chains at the end of the resurgence, and the incidence of measles in China reached its lowest level in history in 2017. 4 out of 31 provinces approached the elimination stage, with low incidence rates of less than one per million population.

With measles SIAs targeting children aged 8 months through 14 years from 2004 to 2010 [9–11], the proportion of reported measles cases in patients within this age range declined gradually from 60% in 2005 [10] to 35% in 2016, which indicated the positive effect of the SIAs. Accordingly, the proportions of cases in infants < 8 months and adults has been increasing in recent years. This finding is consistent with those in other countries, such as Italy, Japan and Portugal. In Italy, Japan and Portugal, the majority of cases occurred among unvaccinated adolescents and young adults [15–17]. These findings indicate that measles elimination presents a major challenge because < 8 months are too young to be vaccinated and adolescent/young adults are not targeted for routine immunization. Although vaccination can decrease the number of cases and control outbreaks, if immunization gaps are not closed and immunization activities do not reach particularly susceptible age groups, cases and outbreaks can occur continually.

Research on the genetic characterization of measles viruses circulating in China from 1993 through 2010 has demonstrated that the genotype H1 was widely distributed throughout the country during this period and that China has a single endemic genotype [8–12, 18–23]. The vast amounts of genetic characterization data obtained in this study allowed us to confirm that H1 is predominant and has been the only endemic virus circulating in mainland China since its initial identification in 1993 [18–23]. Additionally, multiple imported viruses with limited spread have been detected since 2009 [3, 24–30]. This situation is different from that in other countries, such as Italy, the Philippines, Myanmar, Indonesia, and Malaysia, where endemic measles viruses spread with multiple genotypes cocirculating at the same time or with the dominant genotype shifting over time[14, 15, 31]. As of 2017, viruses such as genotypes B3, D8, D9, and D11 have been imported in several provinces in China with limited local transmission [3, 24–30]. In this study, 150 non-H1 viruses representing six genotypes were detected among the extensive background of viruses of the predominant H1 genotype, indicating a very high sensitivity of measles surveillance in China. In countries including Japan, the Republic of Korea, the USA, Canada and Ireland, where endemic transmission of the virus has been interrupted, imported viruses were introduced to the countries with limited transmission [17, 32–36].

Our previous studies divided genotype H1 into 2 clusters, cluster 1 and cluster 2 [19–23]. These two clusters of viruses were cocirculating from 1993 to 2005, whereas cluster 1 has been continuously circulating since 2006 [11, 19–23]. The phylogenetic tree of cluster 1 of H1 viruses showed evidence of multiple chains of transmission during 2011–2017. Although two main lineages in cluster 1 of genotype H1 were identified, no evidence of geographic or temporal restriction was found; identical sequences were detected in multiple provinces at the same time, and identical sequences were sometimes detected in different years in the same province.

Integrated with the molecular epidemiologic data, epidemiological evidence can be used to identify or confirm the interruption of transmission chains. However, in the present study, it was difficult to obtain robust epidemiological evidence for the transmission chains in China, where endemic measles viruses continue to circulate throughout many provinces. Therefore, phylogenetic data were used to investigate the transmission chains of MeV outbreaks in this study.

The phylogenetic analysis of the N450 sequence window indicated that most of the transmission chains detected in the 2011–2012 strains had been interrupted. In addition, many new transmission chains of unknown origin were identified that caused a large measles resurgence in China during 2013–2016. After 4 years of measles resurgence and continuous routine immunization programs and SIAs, only a few strains persisted, being sporadically detected in China in 2017. As part of the measles surveillance system, molecular surveillance is an important tool for monitoring the circulation of wild-type viruses in a region over time. Information on genotype and sequence variants of circulating viruses can help to identify the potential sources of virus importation and recognize long-lasting virus transmission chains [5]. Currently, the N450 sequence window is recommended by the WHO for the genotyping of MeV. However, when viruses from the same genotype are identified among epidemiologically unlinked cases, N gene sequences may exhibit insufficient diversity to allow differentiation between the continuous circulation of MeV and multiple new introductions [5, 37]. Recently, the use of new sequencing windows, including the M-F NCR, has been recommended for improving measles surveillance [37]. The higher variability of this region than of the N450 sequence provides better discrimination of chains of transmission than do the N450 sequences of the MeV genome [37, 38]. However, more sequences from M-F NCR should be obtained from regions worldwide to provide a database to improve the resolution of molecular epidemiology analysis.

The elimination of measles is an important goal for public health worldwide. To ensure the success of measles elimination, a two-dose vaccine regimen with 95% coverage for all population groups and age cohorts is necessary to establish herd immunity and prevent subsequent epidemics [6, 39]. China is currently in the measles pre-elimination phase. The incidence of measles reached the lowest level in history in 2017 and continued to decrease in 2018 (unpublished data). However, measles outbreaks have occurred again in many countries where circulating endemic measles were previously eliminated, such as in the USA, Japan, Mongolia, Brazil and other countries [16, 34, 40–43]. Therefore, measles elimination in China and other countries remains a challenge.

## Conclusion

In this study, by combining analyses of epidemiology data and molecular virological data, we demonstrated the great progress towards measles elimination in China by the further analysis of circulation dynamics for the endemic H1 genotype measles virus from 2011 to 2017. This study provides comprehensive information to the world that can support measles elimination in the future, not only in China but also in other countries worldwide. The information provided can aid other countries in tracing the sources of measles cases and identifying transmission links to help control potential measles outbreaks.

## Supporting information

S1 Table Information on the 152 measles strains used to generate the phylogenetic dendrograms based on the 450-nucleotide sequence of the N gene C-terminal region. (DOCX)

## References

[1] Cutts FT, Markowitz LE. Successes and failures in measles control. J Infect Dis. 1994; 170(1): 32–41. 10.1093/infdis/170.supplement_1.s32 . 7930752

[2] Bellini WJ, Rota PA. Genetic diversity of wild-type measlesviruses: implications for global measles elimination programs. Emerg Infect Dis. 1998; 4:29–35. 10.3201/eid0401.980105 . 9452396 PMC2627654

[3] Zhang Y, Ding Z, Wang H, Li L, Pang Y, Brown KE, et al New Measles Virus Genotype Associated with Outbreak, China. Emerg Infect Dis. 2010; 16:943–947. 10.3201/eid1606.100089 . 20507744 PMC3086224

[4] WHO. Measles virus nomenclature update: 2012. Wkly Epidemiol Rec. 2012; 87(9): 73–81. 22462199

[5] WHO. Genetic diversity of wild-type measles viruses and the global measles nucleotide surveillance database (MeaNS). Wkly Epidemiol Rec. 2015; 90:373–380. 26211016

[6] Kalaycioglu AT, Yolbakan S, Guldemir D, Korukluoglu G, Coskun A, Cosgun Y, et al Towards measles elimination: Phylogenetic analysis of measles viruses in Turkey (2012–2013) and identification of genotype D8. J Med Virol. 2016; 88(11):1867–73. 10.1002/jmv.24548 27089242

[7] WHO. Measles. 2018 11 29 Available from: https://www.who.int/en/news-room/fact-sheets/detail/measles.

[8] Lixia W, Guang Z, Lee LA, Zhiwei Y, Jingjin Y, Jun Z, et al Progress in accelerated measles control in the People's Republic of China, 1991–2000. J Infect Dis. 2003; 187 (Suppl 1):S252–257. 10.1086/368045 . 12721922

[9] Ma C, An Z, Hao L, Cairns KL, Zhang Y, Ma J, et al Progress toward measles elimination in the People’s Republic of China, 2000–2009. J Infect Dis. 2011; 204(Suppl 1):S447–454. 10.1093/infdis/jir103 . 21666198

[10] Ma C, Hao L, Zhang Y, Su Q, Rodewald L, An Z, et al Monitoring progress towards the elimination of measles in China: an analysis of measles surveillance data. Bull World Health Organ. 2014; 92(5):340–347. 10.2471/BLT.13.130195 . 24839323 PMC4007128

[11] Zhang Y, Wang H, Xu S, Mao N, Zhu Z, Shi J, et al Monitoring progress toward measles elimination by genetic diversity analysis of measles viruses in China 2009–2010. Clin Microbiol Infect. 2014; 20(9):O566–577. 10.1111/1469-0691.12530 . 24438091

[12] Xu W, Zhang Y, Wang H, Zhu Z, Mao N, Mulders MN, et al Global and national laboratory networks support high quality surveillance for measles and rubella. Int Health. 2017; 9: 184–189. 10.1093/inthealth/ihx017 . 28582561

[13] China Center for Disease Control and Prevention. National Measles Surveillance Plan (2014). Issued by Dept. of Disease Control and Prevention (2014) No.36, 2014-02-21.

[14] Rota PA, Brown K, Mankertz A, Santibanez S, Shulga S, Muller CP, et al Global distribution of measles genotypes and measles molecular epidemiology. J Infect Dis. 2011; 204(Suppl 1):S514–S523. 10.1093/infdis/jir118 . 21666208

[15] Tramuto F, Maida CM, Pojero F, Colomba GME, Casuccio A, Restivo V, et al Case-based surveillance of measles in Sicily during 2012–2017: The changing molecular epidemiology and implications for vaccine strategies. PLoS One. 2018; 13(4): e0195256 10.1371/journal.pone.0195256 . 29617454 PMC5884552

[16] Komabayashi K, Seto J, Tanaka S, Suzuki Y, Ikeda T, Onuki N, et al The largest measles outbreak, including 38 modified measles and 22 typical measles cases, Yamagata, Japan, 2017 in its elimination era. Jpn J Infect Dis. 2018; 71(6): 413–418. 10.7883/yoken.JJID.2018.083 . 29962488

[17] Augusto GF, Cruz D, Silva A, Pereira N, Aguiar B, Leça A, et al Challenging measles case definition: three measles outbreaks in three Health Regions of Portugal, February to April 2018. Euro Surveill. 2018; 23(28). 10.2807/1560-7917.ES.2018.23.28.1800328 . PMC6152152 30017024

[18] Xu W, Tamin A, Rota JS, Zhang L, Bellini WJ, Rota PA. New genetic group of measles virus isolated in the People's Republic of China. Virus Res. 1998; 54:147–156. . 9696123 10.1016/s0168-1702(98)00020-3

[19] Zhang Y, Zhu Z, Rota PA, Jiang X, Hu J, Wang J, et al Molecular epidemiology of measles viruses in China, 1995-2003.Virol J. 2007; 4:14 10.1186/1743-422X-4-14 . 17280609 PMC1802751

[20] Zhang Y, Ji Y, Jiang X, Xu S, Zhu Z, Zheng L, et al Genetic characterization of measles viruses in China, 2004. Virol J. 2008; 5:120 10.1186/1743-422X-5-120 . 18928575 PMC2600640

[21] Ji Y, Zhang Y, Xu S, Zhu Z, Zuo S, Jiang X, et al Measles Resurgence Associated with Continued Circulation of Genotype H1 Viruses in China, 2005. Virol J. 2009; 6:135 10.1186/1743-422X-6-135 . 19737391 PMC2759936

[22] Ji Y, Xu S, Zhang Y, Zhu Z, Mao N, Jiang X, et al Genetic characterization of wild-type measles viruses isolated in China, 2006–2007. Virol J. 2010; 7:105 10.1186/1743-422X-7-105 . 20500809 PMC2887432

[23] Zhang Y, Xu S, Wang H, Zhu Z, Ji Y, Liu C, et al Single endemic genotype of measles virus continuously circulating in China for at least 16 years. PLoS One. 2012; 7(4): e34401 10.1371/journal.pone.0034401 . 22532829 PMC3332093

[24] Zhang Y, He JL, Sun L, Wang HL, Xu WB. D9 measles virus was first isolated from an imported measles case in Sichuan Province of China. Zhong Guo Yi Miao He Mian Yi. 2009; 15(4):304–309. Chinese. . 20077726

[25] Wang HL, Zheng L, Wang JT, Gao H, Zhang Y, Kong XH, et al The Report on the First Imported The first imported measles case associated with genotype D4 measles virus in China. Bing Du Xue Bao. 2010; 26(2):103–108. Chinese. . 20480638

[26] Sun XD, Li CS, Tang X, Li Z, Zhang Y, Tang W, et al Genetic characterization analysis on the first imported measles virus of genotype D8 in Chinese mainland. Bing Du Xue Bao. 2013; 29(6):583–588. Chinese. . 24520762

[27] Fang X, Sun J, Zhang Y, Wang C, Song Y, Song L, Xu Q, Li B, Chen P, Li H, Xu A. The first measles outbreak caused by imported genotype D9 measles virus in Shandong Province, China, 2013. Jpn J Infect Dis. 2014; 67(4):300–303. . 25056078 10.7883/yoken.67.300

[28] Wang SL, Li CS, Wang HL, Tang W, Song JH, Yang JH, et al Imported B3 Genotype Measles Viruses were Isolated from Measles Cases in the Chinese Mainland. Bing Du Xue Bao. 2014; 30(5):3535–540. Chinese. . 25562963

[29] Li S, Qian X, Yuan Z, Sun X, Li C, Tang X, et al Molecular epidemiology of measles virus infection in Shanghai in 2000–2012: the first appearance of genotype D8.Braz J Infect Dis. 2014; 18(6):581–590. 10.1016/j.bjid.2014.05.018 . 25281832 PMC9425214

[30] Chen M, Zhang Y, Huang F, Wang H, Liu D, Li J, et al Endemic and Imported Measles Virus-Associated Outbreaks among Adults, Beijing, China, 2013. Emerg Infect Dis. 2015; 21(3):477–479. 10.3201/eid2103.140646 . 25695536 PMC4344261

[31] Takashima Y, Schluter WW, KM LM, Diorditsa S, Quiroz Castro M, Ou AC, et al Progress toward measles elimination-Philippines, 1998–2014. MMWR Morb Mortal Wkly Rep. 2015; 64:357–362. . 25856257 PMC4584627

[32] Yang TU, Kim JW, Eom HE, Oh HK, Kim ES, Kang HJ, et al Resurgence of measles in a country of elimination: Interim assessment and current control measures in the Republic of Korea in early 2014. Int J Infect Dis. 2015; 33:12–14. 10.1016/j.ijid.2014.09.016 . 25447718

[33] Rota PA, Liffick SL, Rota JS, Katz RS, Redd S, Papania M, et al Molecular epidemiology of measles viruses in the United States, 1997–2001. Emerg Infect Dis. 2002; 8:902–908. 10.3201/eid0809.020206 . 12194764 PMC2732556

[34] Centers for Disease Control and Prevention. Measles-United States, 2011. MMWR Morb Mortal Wkly Rep. 2012; 61: 253–257. . 22513526

[35] Thomas S, Hiebert J, Gubbay JB, Gournis E, Sharron J, Severini A, et al Measles Outbreak with Unique Virus Genotyping, Ontario, Canada, 2015. Emerg Infect Dis. 2017; 23(7):1063–1069. 10.3201/eid2307.161145 . 28628461 PMC5512469

[36] Barrett P, Cotter S, Ryan F, Connell J, Cronin A, Ward M, et al A national measles outbreak in Ireland linked to a single imported case, April to September, 2016. Euro Surveill. 2018; 23(31). 10.2807/1560-7917.ES.2018.23.31.1700655 . PMC6167609 30086818

[37] Penedos AR, Myers R, Hadef B, Aladin F, Brown KE. Assessment of the Utility of Whole Genome Sequencing of Measles Virus in the Characterisation of Outbreaks. PLoS One. 2015; 10(11):e0143081 10.1371/journal.pone.0143081 . 26569100 PMC4646484

[38] Gil H, Fernández-García A, Mosquera MM, Hübschen JM, Castellanos AM, de Ory F, et al Measles virus genotype D4 strains with non-standard length M-F non-coding region circulated during the major outbreaks of 2011–2012 in Spain. PLoS One. 2018; 13(7):e0199975 10.1371/journal.pone.0199975 . 30011283 PMC6047782

[39] Muscat M, Shefer A, Ben Mamou M, Spataru R, Jankovic D, Deshevoy S, et al The state of measles and rubella in the WHO European Region, 2013. Clin Microbiol Infect, 2014, 20: 12–18. 10.1111/1469-0691.12584 . 24520948

[40] WHO. Measles cases spike globally due to gaps in vaccination coverage. 2018 11 29 Available from: https://www.who.int/news-room/detail/29-11-2018-measles-cases-spike-globally-due-to-gaps-in-vaccination-coverage

[41] WHO. Measles-Madagascar. 2019 1 17 Available from: https://www.who.int/csr/don/17-january-2019-measles-madagascar/en/

[42] Hagan JE, Greiner A, Luvsansharav UO, Lake J, Lee C, Pastore R, etal Use of a Diagonal Approach to Health System Strengthening and Measles Elimination after a Large Nationwide Outbreak in Mongolia. Emerg Infect Dis. 2017; 23(13). 10.3201/eid2313.170594 . 29155667 PMC5711310

[43] Goldani LZ. Measles outbreak in Brazil, 2018. Braz J Infect Dis. 2018; 22(5):359 10.1016/j.bjid.2018.11.001 . 30527066 PMC9427970

