# Supplementary material for: Molecular characterization of measles viruses in China: Circulation dynamics of the endemic H1 genotype from 2011 to 2017
Source: PLoS One. 2019 Jun 20;14(6):e0218782. doi: 10.1371/journal.pone.0218782 (PMC6586441; doi:10.1371/journal.pone.0218782)
Supplement: S1 Table — (DOCX) [file pone.0218782.s001.docx]

S1 Table. Information on the 152 measles strains used to generate the phylogenetic dendrograms based on the 450-nucleotide sequence of the N gene C-terminal region.

| Strain name | Genotype | Collection date | Country | GenBank accession no. |
| --- | --- | --- | --- | --- |
| MVi/Hunan.CHN/20.11/2 | H1 | 17-Jun-2011 | China | MH979841 |
| MVi/Liaoning.CHN/26.11/ | H1 | 25-Aug-2011 | China | MH979842 |
| Mvi/Innermongolia.CHN/25.11/ | H1 | 15-Aug-2011 | China | MH979843 |
| MVi/Sichuan.CHN/15.11/ | H1 | 27-Apr-2011 | China | MH979844 |
| MVi/Shanghai.CHN/36.11/ | H1 | 8-Oct-2011 | China | MH979845 |
| MVi/Tianjin.CHN/11.11/1 | H1 | 31-Mar-2011 | China | MH979846 |
| MVs/Xinjiang.CHN/13.11/ | H1 | 22-Mar-2011 | China | MH979847 |
| MVi/Zhejiang.CHN/25.11/ | H1 | 4-Aug-2011 | China | MH979848 |
| MVi/Anhui.CHN/14.12/ | H1 | 13-Jun-2012 | China | MH979849 |
| MVi/Beijing.CHN/14.12/ | H1 | 6-Aug-2012 | China | MH979850 |
| MVi/Beijing.CHN/18.12/ | H1 | 6-Aug-2012 | China | MH979851 |
| MVs/Guangdong.CHN/20.12/ | H1 | 31-Aug-2012 | China | MH979852 |
| MVi/Henan.CHN/30.12/3 | H1 | 13-Aug-2012 | China | MH979853 |
| MVi/Jiangsu.CHN/16.12/4 | H1 | 9-Aug-2012 | China | MH979854 |
| MVi/Jiangxi.CHN/18.12/ | H1 | 4-Jun-2012 | China | MH979855 |
| MVi/Shanghai.CHN/11.12/3 | H1 | 16-May-2012 | China | MH979856 |
| MVi/Shanghai.CHN/23.12/3 | H1 | 6-Jul-2012 | China | MH979857 |
| MVi/Shanghai.CHN/29.12/2 | H1 | 31-Aug-2012 | China | MH979858 |
| MVi/Shanghai.CHN/30.12/5 | H1 | 31-Aug-2012 | China | MH979859 |
| MVi/Shanghai.CHN/45.12/2 | H1 | 14-Dec-2012 | China | MH979860 |
| MVi/Tianjin.CHN/20.12/ | H1 | 28-Jun-2012 | China | MH979861 |
| MVi/Yunnan.CHN/50.12/2 | H1 | 21-Jan-2013 | China | MH979862 |
| MVi/Anhui.CHN/06.13/7 | H1 | 12-Apr-2013 | China | MH979863 |
| MVi/Anhui.CHN/07.13/5 | H1 | 12-Apr-2013 | China | MH979864 |
| MVi/Anhui.CHN/08.13/9 | H1 | 12-Apr-2013 | China | MH979865 |
| MVi/Beijing.CHN/17.13/5 | H1 | 18-Jun-2013 | China | MH979866 |
| MVi/Beijing.CHN/10.13/6 | H1 | 18-Jun-2013 | China | MH979867 |
| MVi/Chongqing.CHN/12.13/ | H1 | 31-May-2013 | China | MH979868 |
| MVs/Guangdong.CHN/14.13/ | H1 | 29-Apr-2013 | China | MH979869 |
| MVs/Guangdong.CHN/14.13/10 | H1 | 27-May-2013 | China | MH979870 |
| MVi/Gansu.CHN/13.13/4 | H1 | 7-Jun-2013 | China | MH979871 |
| MVi/Guizhou.CHN/11.13/ | H1 | 8-Apr-2013 | China | MH979872 |
| MVi/Guizhou.CHN/17.13/ | H1 | 28-May-2013 | China | MH979873 |
| MVi/Henan.CHN/21.13/ | H1 | 21-Jun-2013 | China | MH979874 |
| MVs/Hunan.CHN/04.13/2 | H1 | 29-Jan-2013 | China | MH979875 |
| MVs/Shandong.CHN/10.13/ | H1 | 15-Apr-2013 | China | MH979876 |
| MVs/Shandong.CHN/34.13/4 | H1 | 21-Nov-2013 | China | MH979877 |
| MVi/Shanghai.CHN/16.13/14 | H1 | 17-May-2013 | China | MH979878 |
| MVs/Anhui.CHN/06.14/ | H1 | 4-Feb-2014 | China | MH979879 |
| MVs/Anhui.CHN/14.14/12 | H1 | 4-Apr-2014 | China | MH979880 |
| MVs/Anhui.CHN/15.14/3 | H1 | 10-Apr-2014 | China | MH979881 |
| MVs/Anhui.CHN/06.14/9 | H1 | 9-Feb-2014 | China | MH979882 |
| MVi/Anhui.CHN/14.14/15 | H1 | 5-Apr-2014 | China | MH979883 |
| MVi/Anhui.CHN/20.14/6 | H1 | 14-May-2014 | China | MH979884 |
| MVi/Anhui.CHN/23.14/2 | H1 | 4-Jun-2014 | China | MH979885 |
| MVi/Anhui.CHN/25.14/ | H1 | 19-Jun-2014 | China | MH979886 |
| MVs/Anhui.CHN/09.14/3 | H1 | 24-Jan-2014 | China | MH979887 |
| MVi/Beijing.CHN/06.14/ | H1 | 6-Feb-2014 | China | MH979888 |
| MVi/Beijing.CHN/21.14/38 | H1 | 22-May-2014 | China | MH979889 |
| MVi/Beijing.CHN/17.14/61 | H1 | 24-Apr-2014 | China | MH979890 |
| MVi/Beijing.CHN/34.14/6 | H1 | 19-Aug-2014 | China | MH979891 |
| MVs/Beijing.CHN/07.14/03 | H1 | 10-Feb-2014 | China | MH979892 |
| MVi/Beijing.CHN/17.14/33 | H1 | 27-Apr-2014 | China | MH979893 |
| MVi/Beijing.CHN/17.14/38 | H1 | 26-Apr-2014 | China | MH979894 |
| MVs/Guangdong.CHN/08.14/6 | H1 | 12-Feb-2014 | China | MH979895 |
| MVi/Guangxi.CHN/14.14/2 | H1 | 3-Apr-2014 | China | MH979896 |
| MVi/Guizhou.CHN/30.14/ | H1 | 24-Jul-2014 | China | MH979897 |
| MVi/Guizhou.CHN/16.14/ | H1 | 20-Apr-2014 | China | MH979898 |
| MVi/Hebei.CHN/01.14/ | H1 | 2-Jan-2014 | China | MH979899 |
| MVi/Henan.CHN/15.14/3 | H1 | 6-Apr-2014 | China | MH979900 |
| MVi/Henan.CHN/22.14/10 | H1 | 29-May-2014 | China | MH979901 |
| MVi/Henan.CHN/27.14/2 | H1 | 29-Jun-2014 | China | MH979902 |
| MVi/Henan.CHN/28.14/2 | H1 | 9-Jul-2014 | China | MH979903 |
| MVi/Henan.CHN/42.14/6 | H1 | 13-Oct-2014 | China | MH979904 |
| MVi/Henan.CHN/44.14/7 | H1 | 27-Oct-2014 | China | MH979905 |
| MVi/Henan.CHN/44.14/10 | H1 | 28-Oct-2014 | China | MH979906 |
| MVi/Henan.CHN/48.14/2 | H1 | 26-Nov-2014 | China | MH979907 |
| MVi/Henan.CHN/51.14/11 | H1 | 14-Dec-2014 | China | MH979908 |
| MVi/Henan.CHN/15.14/2 | H1 | 7-Apr-2014 | China | MH979909 |
| MVi/Henan.CHN/10.14/4 | H1 | 4-Mar-2014 | China | MH979910 |
| MVi/Henan.CHN/09.14/3 | H1 | 26-Feb-2014 | China | MH979911 |
| Mvi Hubei.CHN/32.14/4 | H1 | 6-Aug-2014 | China | MH979912 |
| Mvi Hubei.CHN/15.14/ | H1 | 10-Apr-2014 | China | MH979913 |
| MVi/Hunan.CHN/16.14/ | H1 | 18-Apr-2014 | China | MH979914 |
| MVi/Hunan.CHN/15.14/6 | H1 | 12-Apr-2014 | China | MH979915 |
| MVs/Jilin.CHN/16.14/14 | H1 | 20-Apr-2014 | China | MH979916 |
| MVs/Jilin.CHN/12.14/2 | H1 | 23-Mar-2014 | China | MH979917 |
| MVi/Qinghai.CHN/11.14/ | H1 | 10-Mar-2014 | China | MH979918 |
| MVs/Shandong.CHN/06.14/2 | H1 | 6-Feb-2014 | China | MH979919 |
| MVs/Shandong.CHN/21.14/6 | H1 | 21-May-2014 | China | MH979920 |
| MVs/Shandong.CHN/17.14/16 | H1 | 27-Apr-2014 | China | MH979921 |
| MVs/Shandong.CHN/08.14/12 | H1 | 22-Feb-2014 | China | MH979922 |
| MVs/Shandong.CHN/26.14/4 | H1 | 23-Jun-2014 | China | MH979923 |
| MVs/Shandong.CHN/11.14/3 | H1 | 14-Mar-2014 | China | MH979924 |
| MVs/Shandong.CHN/30.14/6 | H1 | 27-Jul-2014 | China | MH979925 |
| MVs/Shandong.CHN/08.14/14 | H1 | 22-Feb-2014 | China | MH979926 |
| MVi/Shanxi.CHN/22.14/ | H1 | 31-May-2014 | China | MH979927 |
| MVi/Shanxi.CHN/44.14/4 | H1 | 1-Nov-2014 | China | MH979928 |
| MVi/Shanxi.CHN/08.14/1 | H1 | 20-Feb-2014 | China | MH979929 |
| MVi/Shanxi.CHN/08.14/2 | H1 | 19-Feb-2014 | China | MH979930 |
| MVi/Shanxi.CHN/16.14/2 | H1 | 18-Apr-2014 | China | MH979931 |
| MVi/Tianjin.CHN/15.14/16 | H1 | 12-Apr-2014 | China | MH979932 |
| MVs/Yunnan.CHN/10.14/03 | H1 | 8-Mar-2014 | China | MH979933 |
| MVi/Yunnan.CHN/10.14/2 | H1 | 5-Mar-2014 | China | MH979934 |
| MVi/Yunnan.CHN/50.14/3 | H1 | 11-Dec-2014 | China | MH979935 |
| MVi/Zhejiang.CHN/53.14/ | H1 | 30-Dec-2014 | China | MH979936 |
| MVi/Anhui.CHN/2.15/3 | H1 | 6-Jan-2015 | China | MH979937 |
| MVs/Anhui.CHN/10.15/9 | H1 | 2-Mar-2015 | China | MH979938 |
| MVs/Beijing.CHN/4.15/2 | H1 | 23-Jan-2015 | China | MH979939 |
| MVs/Beijing.CHN/13.15/2 | H1 | 24-Mar-2015 | China | MH979940 |
| MVs/Beijing.CHN/9.15/15 | H1 | 22-Feb-2015 | China | MH979941 |
| MVs/Beijing.CHN/15.15/6 | H1 | 10-Apr-2015 | China | MH979942 |
| MVs/Beijing.CHN/21.15/5 | H1 | 18-May-2015 | China | MH979943 |
| MVs/Fujian.CHN/3.15/2 | H1 | 13-Jan-2015 | China | MH979944 |
| MVs/Fujian.CHN/11.15/7 | H1 | 11-Mar-2015 | China | MH979945 |
| MVi/Fujian.CHN/23.15/3 | H1 | 3-Jun-2015 | China | MH979946 |
| MVi/Fujian.CHN/28.15/ | H1 | 9-Jul-2015 | China | MH979947 |
| MVi/Fujian.CHN/29.15/2 | H1 | 18-Jul-2015 | China | MH979948 |
| MVi/Guangdong.CHN/18.15/5 | H1 | 29-Apr-2015 | China | MH979949 |
| MVi/Gansu.CHN/1.15/ | H1 | 8-Jan-2015 | China | MH979950 |
| MVi/Gansu.CHN/12.15/ | H1 | 17-Mar-2015 | China | MH979951 |
| MVi/Gansu.CHN/53.15/2 | H1 | 28-Dec-2015 | China | MH979952 |
| MVi/Henan.CHN/19.15/7 | H1 | 4-May-2015 | China | MH979953 |
| MVi/Henan.CHN/19.15/9 | H1 | 5-May-2015 | China | MH979954 |
| MVs/Henan.CHN/2.15/17 | H1 | 9-Jan-2015 | China | MH979955 |
| MVi/Jiangsu.CHN/12.15/12 | H1 | 19-Mar-2015 | China | MH979956 |
| MVi/Sichuan.CHN/10.15/5 | H1 | 1-Mar-2015 | China | MH979957 |
| MVi/Sichuan.CHN/15.15/24 | H1 | 11-Apr-2015 | China | MH979958 |
| MVi/Sichuan.CHN/21.15/ | H1 | 18-May-2015 | China | MH979959 |
| MVs/Shandong.CHN/4.15/2 | H1 | 21-Jan-2015 | China | MH979960 |
| MVs/Shandong.CHN/9.15/28 | H1 | 25-Feb-2015 | China | MH979961 |
| MVs/Shandong.CHN/13.15/18 | H1 | 28-Mar-2015 | China | MH979962 |
| MVs/Shandong.CHN/14.15/38 | H1 | 4-Apr-2015 | China | MH979963 |
| MVs/Shandong.CHN/16.15/9 | H1 | 12-Apr-2015 | China | MH979964 |
| MVs/Shandong.CHN/52.15/ | H1 | 26-Dec-2015 | China | MH979965 |
| MVi/Shanghai.CHN/20.15/13 | H1 | 14-May-2015 | China | MH979966 |
| MVi/Shanghai.CHN/3.15/3 | H1 | 15-Jan-2015 | China | MH979967 |
| MVi/Shanghai.CHN/11.15/9 | H1 | 2015-03-11 | China | MH979968 |
| MVi/Shanghai.CHN/4.15/2 | H1 | 19-Jan-2015 | China | MH979969 |
| MVi/Shanxi.CHN/3.15/ | H1 | 14-Jan-2015 | China | MH979970 |
| MVs/Yunnan.CHN/21.15/ | H1 | 17-May-2015 | China | MH979971 |
| MVs/Zhejiang.CHN/15.15/10 | H1 | 11-Apr-2015 | China | MH979972 |
| MVi/Zhejiang.CHN/21.15/ | H1 | 17-May-2015 | China | MH979973 |
| MVi/Zhejiang.CHN/13.15/ | H1 | 23-Mar-2015 | China | MH979974 |
| MVi/Anhui.CHN/8.16/4 | H1 | 26-Feb-2016 | China | MH979975 |
| MVs/Beijing.CHN/12.16/15 | H1 | 26-Mar-2016 | China | MH979976 |
| MVs/Beijing.CHN/10.16/11 | H1 | 7-Mar-2016 | China | MH979977 |
| MVi/Gansu.CHN/25.16/ | H1 | 19-Jun-2016 | China | MH979978 |
| MVi/Gansu.CHN/21.16/14 | H1 | 24-May-2016 | China | MH979979 |
| MVs/Henan.CHN/9.16/7 | H1 | 2-Mar-2016 | China | MH979980 |
| MVs/Hubei.CHN/19.16/6 | H1 | 12-May-2016 | China | MH979981 |
| MVs/Hubei.CHN/17.16/3 | H1 | 24-Apr-2016 | China | MH979982 |
| MVs/Shandong.CHN/5.16/8 | H1 | 6-Feb-2016 | China | MH979983 |
| MVs/Shandong.CHN/8.16/19 | H1 | 22-Feb-2016 | China | MH979984 |
| MVs/Shandong.CHN/8.16/38 | H1 | 25-Feb-2016 | China | MH979985 |
| MVs/Shandong.CHN/14.16/37 | H1 | 8-Apr-2016 | China | MH979986 |
| MVi/Tianjin.CHN/14.16/9 | H1 | 8-Apr-2016 | China | MH979987 |
| MVi/Anhui.CHN/17.17/4 | H1 | 27-Apr-2017 | China | MH979988 |
| MVs/Anhui.CHN/12.17/ | H1 | 16-Mar-2017 | China | MH979989 |
| MVs/Beijing.CHN/26.17/ | H1 | 26-Jun-2017 | China | MH979990 |
| MVi/Liaoning.CHN/13.17/ | H1 | 1-Apr-2017 | China | MH979991 |
| MVi/Neimenggu.CHN/35.17/ | H1 | 1-Sep-2017 | China | MH979992 |
